# Are adaptation costs necessary to build up a local adaptation pattern?

**DOI:** 10.1186/1471-2148-9-182

**Published:** 2009-08-03

**Authors:** Sara Magalhães, Elodie Blanchet, Martijn Egas, Isabelle Olivieri

**Affiliations:** 1 Laboratoire de Génétique et Environnement, Institut des Sciences de l'Evolution, Université de Montpellier II, Place Eugène Bataillon Bâtiment 22 cc65, 34095 Montpellier, France; 2 Centro de Biologia Ambiental, Faculdade de Ciências da Universidade de Lisboa, Edificio C2, 30 Piso, Campo Grande, P-1749016 Lisbon, Portugal; 3 Section Population Biology, Institute for Biodiversity and Ecosystem Dynamics, University of Amsterdam, Science Park 904, PO Box 94248, 1090 GE Amsterdam, the Netherlands

## Abstract

**Background:**

Ecological specialization is pervasive in phytophagous arthropods. In such specialization mode, limits to host range are imposed by trade-offs preventing adaptation to several hosts. The occurrence of such trade-offs is inferred by a pattern of local adaptation, i.e., a negative correlation between relative performance on different hosts.

**Results:**

To establish a causal link between local adaptation and trade-offs, we performed experimental evolution of spider mites on cucumber, tomato and pepper, starting from a population adapted to cucumber. Spider mites adapted to each novel host within 15 generations and no further evolution was observed at generation 25. A pattern of local adaptation was found, as lines evolving on a novel host performed better on that host than lines evolving on other hosts. However, costs of adaptation were absent. Indeed, lines adapted to tomato had similar or higher performance on pepper than lines evolving on the ancestral host (which represent the initial performance of all lines) and the converse was also true, e.g. negatively correlated responses were not observed on the alternative novel host. Moreover, adapting to novel hosts did not result in decreased performance on the ancestral host. Adaptation did not modify host ranking, as all lines performed best on the ancestral host. Furthermore, mites from all lines preferred the ancestral to novel hosts. Mate choice experiments indicated that crosses between individuals from the same or from a different selection regime were equally likely, hence development of reproductive isolation among lines adapted to different hosts is unlikely.

**Conclusion:**

Therefore, performance and preference are not expected to impose limits to host range in our study species. Our results show that the evolution of a local adaptation pattern is not necessarily associated with the evolution of an adaptation cost.

## Background

Limits to the host range of an organism may be due to the absence of potential hosts within its geographic range. Alternatively, there may be a cost in adapting to one host that precludes adaptation to other hosts. Compelling evidence of such intrinsic limits to host range within a single species stems from the occurrence of sympatric host races [1-8]. Sympatric host races are detected through molecular markers revealing restricted gene flow among populations inhabiting different hosts and through patterns of local adaptation, i.e., negative correlations between relative performance on different hosts [9,10]. These patterns are thought to reflect a cost of local adaptation: adaptation to one host plant entails reduced performance on another host. This cost may be due to a physiological inability to utilize different hosts, which may be the result of antagonistic pleiotropy between adaptation to different hosts [11] or of the accumulation of deleterious mutations that are only expressed when using some other hosts [12]. The other possibility is that host or mate choice results in individuals remaining on the host-plant they are using, thereby reducing gene flow among populations inhabiting different hosts [13-17]. The physiological cost and host or mate choice are expected to feed back positively into each other, as individuals adapting to one host will tend to prefer that host, and individuals that choose one host or its inhabitants will be exposed to selection more often on that host [9,10,18,19].

Because host races are a snapshot of an evolutionary process that has operated in time, it is difficult to disentangle the relative roles of divergent selection for performance and/or preference in the process of host race formation. Performing experimental evolution enables monitoring populations as they adapt to novel hosts, and hence may help identify the mechanisms limiting the number of hosts that can be colonized. So far, experimental evolution studies have been carried out on the evolution of habitat choice [20,21] and mostly on the consequences of adapting to one host on the performance on other hosts [21-28]. In the present study, we followed the experimental evolution of herbivorous mites facing either one of two novel hosts. By following adaptation in two environments, it is possible to observe the build-up of genotype × environment interactions, which will enable the interpretation of local adaptation patterns [29].

In this paper, we performed experimental evolution of spider mites (*Tetranychus urticae*, Koch) on two novel host plants, in order to follow the process underlying the pattern of genotype × environment interaction. In addition, we tested the occurrence of host and mate choice. Spider mites are known to adapt rapidly to novel hosts [22,24,25,30]. In a previous paper [30], we showed that spider mites from a population that had been on cucumber for more than 400 generations had initially low performance on pepper and tomato. Lines evolving on pepper and tomato during 15 generation had increased oviposition rate and juvenile survival on these novel hosts. Motivated by the mixed results concerning the occurrence of host races between populations inhabiting these hosts in the field [9,10], we extend the analysis of the same selection lines and ask whether:

1/ adaptation detected at generation 15 had further increased by generation 25;

2/ adaptation entailed a cost on the ancestral host or on the alternative novel host;

3/ host choice and mate choice had evolved.

## Results

### Adaptation

**Figure 1:**
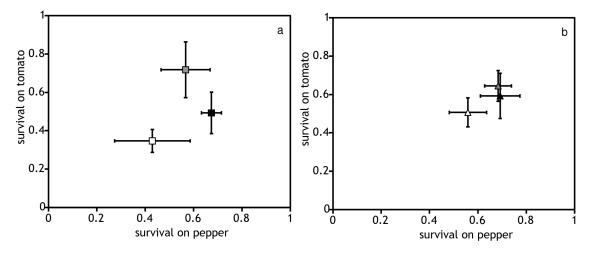
**Juvenile survival, measured as the proportion of individuals surviving to adulthood on the novel hosts at generation 15 (a) and 25 (b)**. White symbols: lines evolving on cucumber, the ancestral host; black symbols: lines evolving on pepper; gray symbols: lines evolving on tomato. Adaptation can be visualized by comparing pepper lines on pepper and tomato lines on tomato to cucumber lines on pepper or on tomato, respectively. The correlated response can be visualized by comparing pepper lines on tomato and tomato lines on pepper to cucumber lines on tomato or on pepper, respectively. Vertical lines correspond to the standard error of the mean, measured as the variation among selection lines of each selection regime.

**Figure 2:**
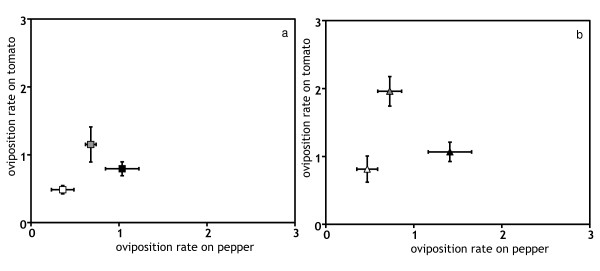
**Oviposition rate, measured as the average number of eggs produced by females during their first 12 days of oviposition at generation 15 (a) and 25 (b)**. Symbols and interpretation as in figure 1.

T-lines had increased juvenile survival and oviposition rate on tomato, as compared to C-lines (Figures 1 and 2, Table 1). Similarly, P-lines had higher values of these traits on pepper than C-lines (Figures 1 and 2, Table 1). Thus, adaptation was detected for these traits on both substrates. Juvenile survival in all populations remained unchanged between generations, as there was no significant effect of the factor generation. The lack of interaction of this factor with the other factors indicates that differences among selection regimes did not change between generations, indicating that adaptation had reached a plateau by generation 15 (Table 1).). Both T and C lines had higher oviposition rates on tomato at generation 25 than at generation 15 (Figure 2a–b), resulting in a significant effect of the factor generation on tomato (Table 1). On pepper no such generation effect was found. The lack of interaction between the generation and selection factors suggests that adaptive changes had also reached a plateau by generation 15.

**Table 1:** Planned comparisons of the GLM to test adaptation and its associated costs.

**Comparison**	**Source**	**Juv. survival**		**Development time**			**Oviposition rate**		**Longevity**		
**Adaptation:**		chisq	*P*	F	Df	*P*	F	*P*	F	Df	*P*
T *vs *C on T	GE	1.429	0.232	1.81	1;8.1	0.215	**13.33**	**0.007**	0.13	1;159	0.721
	SR	**9.712**	**0.002**	2.02	1; 8.6	0.19	**12.61**	**0.007**	1.62	1;40	0.272
	GE*SR	0.462	0.497	0.20	1;7.1	0.666	2.56	0.154	**7.53**	**1;159**	**0.007**
	GE*SL(SR)	0.048	0.827	**41.54**	**8;308**	**<0.0001**	-	-	1.18	3;156	0.319

P *vs *C on P	GE	0.103	0.748	**10.93**	**1;8.9**	**0.009**	2.68	0.136	0.32	1;5.2	0.597
	SR	**10.605**	**0.001**	0.31	1;8.9	0.591	**15.24**	**0.005**	1.34	1;4.1	0.31
	GE*SR	0.521	0.471	0.11	1;7.9	0.746	0.03	0.870	1.55	1; 4.1	0.28
	GE*SL(SR)	0.791	0.374	**12.91**	**8;308**	**<0.0001**	-	-	**2.95**	**5;103**	**0.016**

**Adaptation cost. On ancestral host:**											
T vs C on C	GE	3.173	0.075	**25.1**	**1;8.0**	**0.001**	0.60	0.462	**2.33**	**1;4.0**	**0.201**
	SR	0.257	0.612	0.71	1;7.6	0.426	2.74	0.125	**6.08**	**1;6.4**	**0.047**
	GE*SR	0.079	0.779	1.76	1;7.0	0.226	0.67	0.439	0.60	1;3.09	0.494
	GE*SL(SR)	0.144	0.704	**27.57**	**8;337**	**<0.0001**	-	-	4.23	3;142	0.003
P vs C on C	GE	0.181	0.671	**58.54**	**1;9.1**	**<0.0001**	0.76	0.406	**8.49**	**1;136**	**0.004**
	SR	0.047	0.829	1.33	1;8.1	0.282	0.02	0.892	0.45	1;5.1	0.539
	GE*SR	1.163	0.281	0.44	1;8.1	0.562	0.8	0.398	2.26	1;3.2	0.225
	GE*SL(SR)	0.320	0.572	**8.96**	**9;327**	**<0.0001**	-	-	1.33	3;132	0.268

**On other novel host:**											
T *vs *C on P	GE	0.035	0.851	5.03	1;7.7	0.056	0.45	0.525	2.11	1;4.0	0.22
	SR	2.934	0.087	0.01	1;7.9	0.933	4.05	0.075	0.61	1:3.6	0.483
	GE*SR	0.016	0.90	0.04	1;6.5	0.854	0.75	0.421	0.04	1;2.9	0.857
	GE*SL(SR)	0.003	0.959	**19.18**	**7;302**	**<0.0001**	-	-	**5.21**	**4;114**	**0.001**
P *vs *C on T	GE	2.388	0.122	4.57	1;9.2	0.061	4.2	0.071	0.12	1;30	0.75
	SR	**10.57**	**0.001**	0.00	1;9.0	0.973	3.95	0.082	0.85	1;2.8	0.43
	GE*SR	0.417	0.518	0.12	1;8.1	0.738	0.03	0.862	0.01	1;2	0.922
	GE*SL(SR)	0.890	0.345	**31.84**	**9;277**	**<0.0001**	-	-	**17.35**	**3;129**	**<0.0001**

Juvenile survival: average fraction surviving to adulthood; Development time: days from egg to adulthood; Oviposition rate: mean oviposition rate in the first 12 days; Longevity: average age of death. GE: Generation; SR: Selection regime; SL: selection line. Degrees of freedom given are that of the main effect and of the error, respectively; for fecundity:1;8 in all cases. In bolds are given the effects that are significant at the 0.05 level.

**Figure 3:**
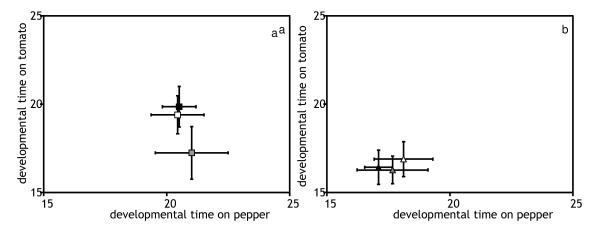
**Developmental time, measured as the day at which females reached adulthood (when they laid their first egg) at generation 15 (a) and 25 (b)**. Symbols and interpretation as in figure 1.

**Figure 4:**
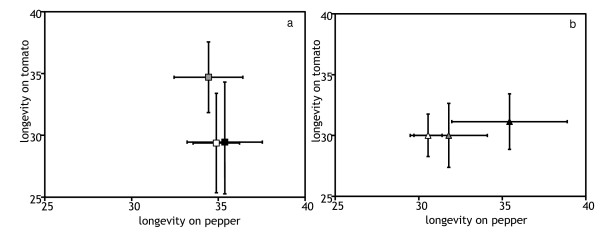
**Longevity, measured as average day of death of the adults, at generation 15 (a) and 25 (b) **Symbols and interpretation as in figure 1.

The development time and the longevity of P- and T-lines on their respective selection substrate were not significantly different from that of C-lines (Figures 3 and 4, Table 1). Hence, these traits did not show adaptive changes (Table 1). There was a significant effect of the generation on pepper, as both C- and P- lines had longer developmental times at generation 25. The interaction between selection line and generation was significant in both comparisons, indicating that lines within each selection regime responded differently in the two generations. For longevity on tomato, a significant selection regime * generation interaction was detected. Indeed, T- lines had a higher longevity on tomato than C lines at generation 15, but not at generation 25. Hence, initial adaptation was lost by generation 25. The interaction between generation and selection line was also significant, indicating that this response varied among lines. No other significant effects were detected.

### Correlated responses

#### (a) On the ancestral host

On cucumber, no significant effect of the selection regime or of its interaction with generation was detected for any trait but longevity for T-line, indicating that mites from either selection regimes overall performed similarly on the ancestral host in both generations (Tables 1 and 2). Therefore, adaptation on tomato and pepper did not entail any cost on the ancestral host. Juvenile survival was marginally affected by the factor generation (Table 1; *P *< 0.1). A significant effect of the generation was also detected for developmental time in the comparison of both P- and T-lines *vs *C-lines, and for longevity in the comparison of P- *vs *C-lines. Indeed, the developmental time on cucumber of mites from all lines was shorter at generation 25 than at generation 15, and the longevity of P- and C-lines increased at generation 25 relative to generation 15 (Table 2). These differences among generations could be due to differences in the environmental conditions between measures.

**Table 2:** Life-history traits on cucumber of mites from the experimental populations evolving on cucumber, pepper and tomato.

**Trait**	**Experimental populations on**		
	***Cucumber***	***Pepper***	***Tomato***
Survival	0.79 ± 0.04	0.78 ± 0.06	0.72 ± 0.04

Development	16.56 ± 0.23	16.4 ± 0.19	17.02 ± 0.37

Oviposition	4.1 ± 0.32	4.49 ± 0.37	3.96 ± 0.21

Longevity	33.45 ± 0.45	34.4 ± 3.14	33.06 ± 2.78

Given are the means ± the s.e.m. of populations evolving on the same host-plant species. No significant differences were found among selection regimes.

#### (b) On the alternative novel host

The traits that had shown significant adaptive changes (juvenile survival and oviposition rate) also increased on the alternative novel host (Figures 1 and 2). Thus, adaptation to one host entailed a positive correlated response on the alternative host. However, this response was only significant for juvenile survival of the P-lines on tomato, all other responses were only marginally significant (*P *< 0.1 in all cases, Table 1). Development time and longevity were not affected by the factor selection regime and no trait was affected by the factor generation (Figures 3 and 4, Table 1). However, the effect of generation was marginally significant for the development time in both comparisons (T-line and P-line compared to ancestral C-line on their respective alternative new host). A significant interaction between lines and generation was detected for development time and longevity in all cases (Table 1).

### Is there a pattern of local adaptation?

To test for the occurrence of a local adaptation pattern, we compared the traits that had evolved in the T- and P-lines on tomato and pepper. There was no difference between selection regimes for juvenile survival (on pepper: χ_1_= 1.36, *P *= 0.24; on tomato: χ_1 _= 0.45, *P *= 0.51). However, P-lines had a significantly higher fecundity than T-lines on pepper (F_1 _= 6.30, *P *= 0.03), and a significantly lower fecundity than T-lines on tomato (F_1 _= 9.45, *P *= 0.01). Hence, a pattern of local adaptation was observed for fecundity but not juvenile survival.

### Host and mate choice

**Figure 5:**
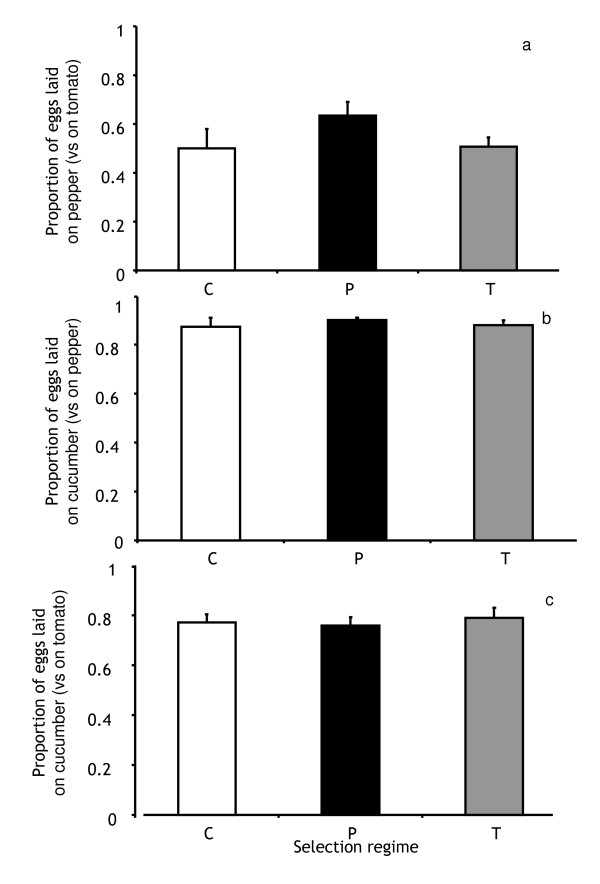
**Host choice of cucumber, pepper and tomato lines (white, black and grey bars, respectively) between (a) pepper and tomato, (b) cucumber and pepper and (c) cucumber and tomato**. The figures show the average proportion of eggs laid on each substrate over four days. Vertical lines correspond to the standard error of the mean, measured as the variation among selection lines of each selection regime.

When given the choice between pepper and tomato, no difference in host choice was found between T- and C-lines (Figure 5a, z = -0.7453, *P *= 0.456) or between P- and C-lines (Figure 5a, z = 1.456, *P *= 0.145). Thus, overall, no evolution of host choice between pepper and tomato was detected on P-lines and T-lines.

When given the choice between cucumber and pepper, mites from all selection regimes laid most of their eggs (between 87 and 98%) on cucumber (Figure 5b; P- *vs *C-lines: z = -1.384, *P *= 0.166; T- *vs *C-lines: z = -0.659, *P *= 0.51). Therefore, this host choice did not evolve in P- and T-lines. Similarly, when given the choice between cucumber and tomato, mites from all selection regimes laid on average 76 to 79% of their eggs on cucumber (Figure 5c; P- *vs *C-lines: z = 1.246, *P *= 0.213; T- *vs *C-lines: z = 0.601, *P *= 0.548). Therefore, this host choice did not evolve in P- and T-lines.

In conclusion, lines from all selection regimes retained a strong preference for their ancestral host, cucumber, and they did not discriminate between pepper and tomato (except P-lines, showing a weak preference for pepper). To assess whether these choices were adaptive, i.e., if mites preferred the host where they performed best, we compared juvenile survival and oviposition rate of lines from the same selection regime on all hosts. All lines survived better on cucumber than on the novel hosts (Figure 1, Tables 2 and 3). Similarly, the oviposition rate of all lines was higher on cucumber than on the novel hosts (Figure 2, Table 2 and Table 4)). Therefore, the host choice between ancestral and novel hosts observed in mites from all selection regimes was adaptive. Concerning differences in performance on pepper and tomato, survival of both T- and C-lines did not differ between these host plants (Figure 1, Table 3), whereas P-lines survived better on pepper than on tomato, although this difference was only marginally significant (Table 3). Differences between oviposition rates on tomato and pepper were found for all lines (Table 4). Indeed, C- and T- lines had higher oviposition rate on tomato than on pepper, whereas the reverse was true for the pepper lines (Figure 2b). Hence, lack of choice observed between the pepper and the tomato substrates does not seem to be adaptive. Possibly, it is associated to the lack of genetic variation for this choice previously found [30].

**Table 3:** Results of the comparisons of juvenile survival of lines from each selection regime on each host.

**Selection regime**	**Source**	**On cucumber vs On pepper**		**On cucumber vs On tomato**		**On tomato vs On pepper**	
		chisq	*P*	chisq	*P*	chisq	*P*

Cucumber	Substrate	11.66	**0.0006**	15.616	**<0.0001**	0.696	0.404
	Line	0.186	0.667	0.017	0.897	0.042	0.837
	Line*substrate	0.002	0.963	0.031	0.861	0.035	0.851

Pepper	Substrate	11.448	**0.0007**	**20.193**	**<0.0001**	3.517	0.061
	Line	10.043	**0.002**	**4.223**	**0.040**	**5.461**	**0.019**
	Line*substrate	2.951	0.086	0.709	0.400	2.707	0.100

Tomato	Substrate	**7.140**	**0.008**	**10.59**	**0.001**	0.481	0.489
	Line	0.865	0.352	0.508	0.476	0.046	0.830
	Line*substrate	0.238	0.238	0.196	0.896	0.059	0.808

**Table 4:** Results of the comparisons of oviposition of lines from each selection regime on each host.

**Selection regime**	**Source**	**On cucumber vs On pepper**		**On cucumber vs On tomato**		**On tomato vs On pepper**	
		F	*P*	F	*P*	F	*P*

Cucumber	Substrate	1208.10	**<0.0001**	61.32	**0.014**	1625.61	**<0.0001**
	Line	0.400	0.805	0.24	0.903	1.31	0.270
	Line*substrate	1.99	0.099	4.15	**0.0033**	1.14	0.341

Pepper	Substrate	93.61	**0.0006**	195.10	**<0.0001**	117.93	**<0.0001**
	Line	0.99	0.505	5.43	**0.0004**	0.96	0.515
	Line*substrate	11.15	**<0.0001**	1.45	0.220	13.92	**<0.0001**

Tomato	Substrate	125.83	**0.0004**	19.21	**0.012**	146.05	**0.0003**
	Line	0.92	0.530	0.50	0.741	0.84	0.565
	Line*substrate	12.34	**<0.0001**	7.53	**<0.0001**	13.98	**<0.0001**

Female mites from all selection regimes did not show a significant preference for males from either tomato or pepper (C-lines: 58%, Gp = 1.29, *P *= 0.26; T-lines: 54%, Gp = 0.08, *P *= 0.78; P-lines: 52%, Gp = 0.32, *P *= 0.57). No heterogeneity was found among lines from the same selection regime (C-lines: Gh= 2.81, *P *= 0.6; T-lines: Gh = 1.128, *P *= 0.89; P-lines: Gh = 0.48, *P *= 0.98). Similarly, no significant discrimination was observed between males from pepper and males from cucumber (C-lines: 43%, Gp = 0.964, *P *= 0.326; T-lines: 53%, Gp = 0.105, *P *= 0.746; P-lines: 60%, Gp = 2.014; *P *= 0.156). This response was not heterogeneous within C- and T-lines (Gh = 3.788, *P *= 0.435 and Gh = 5.618, *P *= 0.23, respectively), but P-lines showed heterogeneous responses (Gh = 10.357, *P *= 0.035). Finally, no preference was found between males from cucumber and males from tomato, although females from tomato showed a marginally significant preference for males from cucumber over males from tomato (C-lines: 60%, Gp = 2.014, *P *= 0.156; T-lines: 65%, Gp = 3.816, *P *= 0.051; P-lines: 54%, Gp = 0.31, *P *= 0.58). No heterogeneity was found within C- and P-lines (Gh = 2.647, *P *= 0.619 and Gh = 3.89 *P *= 0.422, respectively), but it was present in T-lines (Gh = 9.56, *P *= 0.048).

## Discussion

Adaptation of spider mites to tomato and pepper occurred within 15 generations through increased juvenile survival and oviposition; these traits did not increase further by generation 25. Adaptation did not entail a cost: performance on the ancestral host (cucumber) was similar between lines evolving on that host and lines evolving on the novel hosts. Even though mites evolving on a given novel host performed better on that host than mites selected on another novel host (a local-adaptation pattern), adaptation to one host entailed a neutral or a positive response on the alternative novel host. All mites preferred the ancestral host over each of the novel hosts. This choice was adaptive, as traits that had shown adaptive changes still had higher values on the ancestral than on the novel hosts for all lines from all selection regimes. Mites did not discriminate between tomato and pepper in host choice tests. There was no evidence for mate choice between males (or for a competitive advantage of males) from different selection regimes.

The rapid adaptation to novel hosts was not accompanied by any measurable cost on the ancestral host. Assuming trade-offs in host exploitation, costs of adaptation to a novel host should be expressed as reduced performance on the ancestral host. The fact that our data do not show such costs may indicate that most alleles conferring adaptation on tomato or on pepper but having an antagonist pleiotropic effect on cucumber have disappeared from the base population due to purifying selection. Alternatively, it could be that different sets of genes confer adaptation to the novel hosts and to the ancestral host in our population, i.e., trade-offs in host exploitation are absent.

Pioneering studies with the mite species used in this study have tested the occurrence of adaptation costs [22,24,25]. Despite being frequently invoked as examples supporting an adaptation cost [29,31-35], we believe that in fact these studies did not clearly reveal such costs. First, when compared to the ancestral populations, mites selected on a novel host did not show any loss of adaptation to the ancestral host (Table 1 in Fry, 1990 [24]; page 795 in Gould 1979 [25]), and sometimes showed the reverse pattern (Figure 2 in Agrawal, 2000 [22]). Second, these authors created reversion lines, that is, lines that evolved first on a novel host and subsequently evolved on the ancestral host again. As acknowledged by Fry (1990) [24], to support the existence of an adaptation cost, the performance of reverted lines on the novel host must be assayed at the time of reversion, and this value should then be compared to that obtained after some generations of reversal, under the same environmental conditions. This test was not done by either Agrawal (2000) [22] or Gould (1979) [25]. Furthermore, as also acknowledged by Fry (1990) [24], because environmental (experimental) conditions are typically variable, any temporal change in absolute fitness can be due to either a genetic change, an environmental change, or a combination of both. Under the null hypothesis that reversion lines did not experience any loss of fitness on the novel host after reversion, the slope of the curve linking performance at the time of reversion to performance at the time of the assay, should not significantly differ between control lines and reversion lines. Such a test was not performed in any of the previous studies, although Fry's (1990) [24] analysis does suggest that his results were not due to the continuation of the adaptation process on the novel host for a longer period of time in non reverted lines. Therefore, no study involving spider mites showed an adaptation cost in a clear way.

A lack of cost has been detected in many other studies involving various other organisms [36-40], but other studies did find such costs [41-45]. It is as yet unclear why such variation exists. Some studies have found that the degree of resemblance among environments affects the likelihood of finding a cost [41,46]. However, this likelihood may also hinge on characteristics pertaining to the individuals, such as complexity or the mating system. For example, it may well be that costs of adaptation are less frequent when adaptation stems from the standing genetic variation present in a population, which is likely to be the case here. This is because alleles from the standing genetic variation were exposed to selection, hence purging of deleterious alleles has left only the alleles that are globally beneficial. In addition, as both tomato and pepper are within the range of hosts of spider mites, the alleles that increased in frequency may have been already selected on these host plants prior to the 400 generations they spent on cucumber.

Adaptation to the novel hosts was neutral in most cases, *i.e*., evolution on a given host was not accompanied by a correlated response on the alternative host, suggesting that different sets of genes were involved in the adaptation to either tomato or pepper. For juvenile survival of tomato lines, however, adaptation to tomato was accompanied by an increased performance on pepper. Such synclinal selection [45] may lie with the fact that both plants are Solanaceae, hence they might require a similar set of adaptations. Surprisingly, positive genetic correlations among life-history traits on the two host plants were not detected in the base population used to create these selection lines [30].

Assuming some overlap between genes determining adaptation to pepper and tomato, we would have expected that, after an initial phase where performance would have increased on both novel hosts (*i.e*., positively correlated response), in a second phase the increase of performance on one host would have been accompanied by a decreased performance on the alternative host (*i.e*., negatively correlated response). That is, we would expect some cost of adaptation to build up, when alternative alleles would be involved in adaptation to alternative hosts. We did not observe any such negatively correlated response. Hence, as with performance on the ancestral host, we did not find any costs of adaptation in performance on the alternative novel host.

It could be that the period of experimental evolution was too short for costs of adaptation to arise. However, the adaptive response reached a plateau by generation 15, suggesting that most adaptive changes had occurred within the experimental period (but see [47]). This decreases the probability of finding costs later on. Therefore, our results indicate that adaptation to each host is, at least to a large extent, determined by independent loci. Another possibility is that trade-offs do exist, but between traits that we have not measured. However, we measured all traits that are relevant for the intrinsic growth rate of a population, hence if trade-offs exist between traits, they are not translated into differences in performances.

Despite the occurrence of synclinal selection, experimental evolution still resulted in a local adaptation pattern, especially in the oviposition rate. Indeed, pepper lines had higher oviposition rate on pepper than tomato lines, and the reverse was true on tomato. Several studies predict that this pattern is sufficient to restrict gene flow among populations locally adapted to alternative hosts [18,29,48,49]. Indeed, if a given host plant is already used by a locally-adapted population, migrants with a lower fitness on that host will be outcompeted. This will reduce gene flow among populations inhabiting different hosts, thereby fostering local adaptation. In our study, if individuals adapted to pepper would migrate to tomato plants that had been colonized for 15 generations, they would be outcompeted by these local populations. However, if tomato plants were not occupied, pepper populations would not have any intrinsic limitations in their ability to colonize that host. Hence, pre-existing specialization could be reinforced, but this would not prevent colonization of novel, unused hosts.

Theoretical models of host specialization usually predict that the co-occurrence of adaptation and adaptive host preference favours the evolution of specialization [50-52]. These models work under the assumption that herbivores compete for the plant resources they adapt to. Thus, the host choice measured in experimental evolution set-ups, which typically exclude foreign competitors, can only relate to theoretical predictions whenever both the "home vs away" criterion (i.e., individuals perform better in their own environment than in other environments) and the "local vs foreign" criterion for local adaptation (i.e., individuals perform better in their own environment than competitors from other environments) are met [53]. In our experiment, mites evolving on novel hosts still performed better on the ancestral host than on each of the novel hosts. Hence, adaptation did not modify host ranking with regards to performance. Because our experimental setup excluded competition, and given that mites can choose host plant according to their quality [54], adaptive choices are expected to be based on differences in trait values of mites on the different host plants, which corresponds to the "home vs away" criterion for local adaptation. Therefore, in all our selection lines, a preference for the ancestral host is expected. This is indeed what we found: mites always showed a strong preference for the ancestral host, irrespective of whether the alternative choice was tomato or pepper. Alternatively, it could be that no genetic variation for host choice between ancestral and novel hosts was present in the base population, as it was the case for the choice between pepper and tomato [30].

In contrast to predictions concerning host choice, predictions concerning mate choice or male competitive advantage should be based on the relative performance of the potential mates on the host where the offspring will develop, which satisfies the "local vs foreign" criterion. In our experiment, there was no effect of selection regime on male access to females. It is possible that females did not have access to the cues necessary to perform a choice. For example, mate choice was assessed on a neutral substrate, and mites from different selection regimes had a common diet. Both the substrate and the diet of the individual in an interaction are cues known to be used by several arthropods [55].

## Conclusion

In this study, a population of cucumber-adapted spider mites adapted to novel host plants over the course of 15 generations. Hence, the host range of this population was expanded within a short timeframe. Overall, our results suggest that there are as yet no intrinsic limits to spider mites being generalists. Thus, the occurrence of host races [6,10,56] cannot at present be explained by limited phenotypic plasticity or by strong genetic trade-offs in adaptation to different host plants. It is possible, however, that on a larger time scale, genes with antagonistic pleiotropy become involved in the adaptation process or that mutations that are deleterious in the alternate environment accumulate. The other possibility is that host races form because mites from populations established on a given host outcompete migrants from other hosts. Hence, we predict that the occurrence of limits to host range in spider mites necessitates periods of isolation on different host plants. Moreover, our results suggest that competition, rather than host-related adaptations, may be an important mechanism limiting gene flow among populations.

## Methods

Stock cultures and selection lines were done as described in [30]. Briefly, a laboratory-adapted spider mite (*Tetranychus urticae*) population that has been on cucumber for more than 400 generations was used to create selection lines on the host plants cucumber (*Cucumis sativus*), tomato (*Lycopersicon lycopersicum*) and pepper (*Capsicum annuum*, There were five selection lines for each plant species (hereafter called "selection regimes"), each starting with 300 adult females. A previous study [30] showed that (1) there was some genetic variation in the base population for survival, fecundity and longevity on both tomato and pepper, but not for developmental rate on both plants, (b) survival and fecundity on both plants increased after 15 generations of selection and (c) there was a positive correlation between fecundity, longevity and juvenile survival in each environment but no genetic correlation between traits on the two host plants.

### Life-history traits

Experiments were performed in an acclimatized room at approximately 25°C. Mites from all selection regimes spent one generation on bean, to remove all environmental effects from the selection environment. Bean is a highly suitable host plant for mites from all selection regimes, as juvenile survival is nearly 100% and the oviposition rate of females is very high (S. Magalhães, pers. obs.). Life-history traits of mites from each selection line were measured on detached leaves of the three host plant species (cucumber, tomato and pepper), placed on water-soaked cotton wool inside a plastic tray (20*10*5 cm). Development time and survival to adulthood were assessed by transferring eggs laid on that day to leaves of each plant. There were 2–5 cohorts per line. Every four days during the first 12 days and every other day thereafter, we recorded the individuals that died, drowned or became adults. Subsequently, mated females of each selection line were placed on a separate leaf and oviposition rate was measured by counting the eggs laid by all females every three days during 12 days (10–40 females per population). Leaves were replaced after each counting and dead females were discarded. Five selection lines were used for the cucumber and the pepper selection regime (hereafter C- and P-lines, respectively), and four for the Tomato selection regime (hereafter T-lines). For three P-, T- and C-lines, except C-lines on Tomato (two lines), the experiment was prolonged until all females died, to assess total fecundity and longevity. Traits were measured approximately at 8 and 12 months after initiating the selection lines, which roughly corresponds to generations 15 and 25, respectively. The procedure for the life-history traits at generation 25 differed from that at generation 15 described above in that (a) the measure of fecundity was based on individual fecundity of 10 to 20 females per population and (b) five lines per selection regime were used in all cases.

### Host choice and mate choice

Host choice and mate choice was measured at generation 25. Females from each selection line were put on bean to oviposit for 24 hours, to create a cohort. When juveniles emerging from those eggs reached the adult female stage, they were placed on a tiny plastic roof between two flanking half-discs of different host plants (tomato and pepper, tomato and cucumber, or pepper and cucumber, diam. 1.5 cm). Leaf discs were placed on water-soaked cotton wool in a plastic tray (20*10*3 cm; 6 double half-discs per tray). Their position was randomized, and the position of the trays was shuffled regularly to homogenize the possible effects of environmental heterogeneity. The number of eggs laid by each female on each plant type was scored every 24 hours during 4 days. Leaf discs were replaced after two days and the female was put back on the bridge. There were 14 to 20 females per line per choice (average: 16.5). The choice between pepper and tomato on the first day of choice has been published previously [30].

To measure mate choice, we followed the procedure described in Vala et al. (2004)[57]. We concentrated on female choice, for which the set-up has been validated [57]. Females from each selection line were put on bean to oviposit for 24 hours. The cohort emerging from those eggs laid eggs on bean as well. When those individuals reached their last moult before adulthood, they were isolated to prevent mating before the experiment. Each replicate consisted of five females from the same selection line on a small piece of bean leaf (approximately 1 cm^2^), together with two males from different selection regimes. Males were marked dorsally with powder of different colors, randomized between replicates. Leaf discs were observed under the microscope until the first mating occurred, which happened within 5 minutes in all replicates. There were 10 replicates per selection line. Note that this experimental set-up can also be viewed as a test for competition between two males of different origins.

### Statistical analysis

The analysis of juvenile survival was performed with a survival analysis using the PHREG procedure in SAS. This analysis uses Cox's proportional hazard model, which allows the inclusion of censored data obtained in time-dependent experiments, to create a regression for the data belonging to each factor. Statistical differences between the regression coefficients were assessed with the Wald test. All other traits (developmental time, oviposition rate and longevity) were tested with General Lineal Models using the GLM procedure in SAS. Analyses were performed on each substrate, as the relevant comparisons involved differences in trait values of individuals from different selection regimes on a particular substrate. The factors of the model were "generation", "selection regime", a factor "selection line" nested into the factor "selection regime", and the interactions "generation * selection regime" and "generation * selection line". In the GLM procedures, the factor "selection line" and its interactions with other factors were included as random factors. Oviposition rate was square-root transformed to comply with the ANOVA assumptions and data from generation 25 was averaged over all females of the same selection line. For this trait, the factor selection line and its interactions were absent from the model. The main factor "selection regime" was tested against the nested factor "selection line", "generation" was tested against the interaction "generation*selection regime", and this interaction was tested against "generation * selection line". The interaction term with the largest *P*-value (among those not significant at the 10% level), was dropped from the analysis and included in the error term [58]. The analysis was performed again, until all remaining interaction terms were significant at the 10% level.

Because C-lines, selected on the ancestral host, represent the ancestral state of the population, testing adaptation and its consequences was done by comparing the performance of lines selected on novel hosts to those lines. To test whether adaptation occurred, we compared trait values on each novel host of lines selected on that host to C-lines. To test whether adaptation entailed a fitness cost on the ancestral host, we compared life-history traits of P- and T-lines to C-lines on cucumber. Correlated responses were assessed by comparing trait values of P-lines to C-lines on tomato, and trait values of T-lines to C-lines on pepper. Lower trait values of P- or T-lines than C-lines would indicate a negative correlated response. To test whether a pattern of local adaptation had emerged between tomato and pepper lines on these hosts, we compared the relative performance of these two selection regimes on tomato and pepper.

The analysis of host choice was done on the proportion of eggs laid by each female on each leaf disc, after arc sine square-root transformation. We tested differences in host choice between selection regimes using a generalized lineal mixed-effects model, with *Selection regime *as a fixed effect, and *Selection line *as a random effect, using the *lmer *function in the lme4 package in R [59]. We assumed a binomial error structure and a logit link between the response variables and the linear combination of the explanatory variables. The significance of *Selection regime *effect was assessed by comparing the described model with and without the *Selection regime *effect using chi-square tests. All models were fitted using unrestricted maximum likelihood (method = ML). A significant intercept indicates that results deviate significantly from 50%, meaning that one substrate is preferred over the other.

To test whether host choice was adaptive, i.e., whether mites preferred the host plant where they performed better, we compared traits values of lines from the same selection regime on each host. We analyzed only juvenile survival and oviposition rate, which had shown adaptive changes, again using SAS. This analysis was done as described above, but with line, substrate and their interaction as factors. Differences in performances between substrates were then analyzed by pairwise comparisons between substrates.

To analyze differences in mate choice, we used the replicated Goodness-of-fit G-test [58]. Because this test does not consider a hierarchical data structure, we performed the test for each selection regime separately. In this analysis, a significant value for the heterogeneity test parameter Gh (tested against a χ^2 ^distribution with 4 degrees of freedom) indicates significant differences among selection lines, and a significant Gp, testing the pooled effect of treatment against a χ^2 ^distribution with 1 degree of freedom, indicates that mites from all lines significantly preferred males from one selection regime over the other.

## Authors' contributions

SM and IO conceived the study and analyzed the data, SM, EB, IO and ME performed the experiments, SM, EB, ME and IO wrote the manuscript. All authors read and approved the final manuscript.
